# Physical, chemical, and microbiological stability study of diluted atropine eye drops

**DOI:** 10.1186/s40780-019-0154-2

**Published:** 2019-12-05

**Authors:** Jumpei Saito, Hitomi Imaizumi, Akimasa Yamatani

**Affiliations:** 1 Department of Pharmacy, National Center for Child Health and Development, 2-10-1 Okura, Setagaya-ku, Tokyo, 175-8535 Japan; 2 Division of Clinical Pharmacology and Oral Formulation Development, National Center for Child Health and Development, 2-10-1 Okura, Setagaya-ku, Tokyo, 175-8535 Japan

**Keywords:** Pediatric, Atropine, Eye drops

## Abstract

**Background:**

Atropine eye drops are indicated for juvenile myopia progression, cycloplegia, amblyopia, and strabismus. According to the package insert, 10 mg/mL atropine eye drops must be diluted for pediatric patients to prevent systemic adverse effects. Compounding units in hospital pharmaceutical departments or community pharmacies are compelled to prepare this essential medication; however, validated atropine stability data is limited and the shelf life after preparation is extremely short. As it is a long-term treatment, a longer shelf life is necessary to improve patient care. This study aimed to demonstrate the physical, chemical, and microbiological stability of diluted atropine eye drops over a period of six months.

**Methods:**

Preparation consists of dilution of a 10 mg/mL atropine solution (Nitten Atropine Ophthalmic Solution 1%; Nitten Pharmaceutical Co., Ltd.) in 0.9% NaCl to concentrations of 0.1, 1.0, 2.5, and 5.0 mg/mL, followed by a sterilizing filtration procedure and then an aseptic filling process of 5 mL in 5 mL polyethylene eyedropper bottles. The entire process is carried out in an overpressure isolator. All concentration products were kept for six months at 25 °C or 5 °C. Visual inspection was conducted and pH, osmolality, and atropine concentration were measured at day 0, day 14, day 28, and every month until six months. Atropine concentration was measured using liquid chromatography tandem mass spectrometry. The sterility was monitored using a method adapted from the Japanese Pharmacopoeia sterility assay.

**Results:**

Atropine remained within ±5% of the target value in the six batches. Osmolality (285 mOsm/kg) as well as pH (5.88) were kept constant. No variations in solution characteristics (crystallization, discoloration) were noted. Sterility was maintained.

**Conclusions:**

This study validated the physical, chemical, and microbiological stability of 0.1, 1.0, 2.5, and 5.0 mg/mL atropine sulfate eye drops conserved inside polyethylene eyedroppers for six months at 25 °C or 5 °C.

## Background

Atropine is a nonselective muscarinic receptor antagonist, and atropine eye drops are frequently used for retardation of progressive myopia in children [1, 2]. Results from research have demonstrated that a low concentration of atropine is useful in retarding myopia progression in a certain proportion of myopic schoolchildren. Atropine is also used for pediatric patients with cycloplegia, amblyopia, and strabismus. Treatments with higher concentrations of atropine eye drops are associated with a higher rate of dropout, a higher level of photophobia due to larger pupil size, and a lower amplitude of accommodation [2, 3]. Dilution is necessary for treatment of pediatric patients. According to a previous report from Japan, except for patients under two years old, 1% eye drops showed a higher incidence rate of side effects than 0.5 and 0.25% eye drops [4].

In Japan and other countries, low-concentration atropine eye drops are not available as a licensed product. According to the package insert, 10 mg/mL atropine eye drops must be diluted for pediatric patients to prevent systemic adverse effects. To adjust the concentration, a self-dilution method is mandatory and compounding units in hospital pharmaceutical departments or community pharmacies are compelled to prepare this essential medication. However, long-term stability and sterility after preparation are not guaranteed. Additionally, there is the risk of inaccurate dosing.

For long-term treatment, a longer shelf life is necessary to improve patient care. This study aimed to demonstrate the physical, chemical, and microbiological stability of diluted atropine eye drops over a period of six months.

## Methods

### Preparation and conservation of diluted atropine sulfate solutions

A total of 100 mL of 0.1, 1.0, 2.5, and 5.0 mg/mL solutions of atropine sulfate were prepared by diluting 1, 10, 25, and 50 mL of 10 mg/mL atropine sulfate solution (Nitten Atropine Ophthalmic Solution 1%; Lot number L1779K; expiration August 2020; Nitten Pharmaceutical Co., Ltd., Nagoya, Japan) in 99, 90, 75, and 50 mL of isotonic sodium chloride solution (0.9% NaCl; Hikari Pharmaceutical Co., Ltd., Tokyo, Japan) to obtain 0.1, 1.0, 2.5, and 5 mg/mL solution (0.01, 0.1, 0.25, and 0.5% atropine sulfate solution). These solutions were sterilely dispensed at 5 mL per one bottle into sterilized white opaque polyethylene eyedropper squeezable bottles (Lot number 344161 J109; MI Chemical Co., Ltd., Hyogo, Japan) using an electron beam sterile syringe equipped with a 0.22 μm pore size filter (Millex-GS; Lot number R8JA9816; Millipore, Darmstadt, Germany) in a laminar air flow microbiological safety cabinet.

### Study design

The stability of 0.1, 1.0, 2.5, and 5.0 mg/mL atropine solutions in multi-dose eyedropper bottles at 25 °C as a typical room temperature condition and 5 °C as a refrigerated condition was assessed. The storage period was set at six months in order to ensure the longer usable time until the next medical examination.

### Stability of diluted atropine sulfate solution in multi-dose eyedroppers

All atropine solutions were stored in a refrigerator (Sanyo Electric Co., Ltd., Osaka, Japan) temperature controlled at 5 °C ± 1 °C or in a highly reliable accuracy oven (Fine Oven; Yamato Scientific Co., Ltd., Tokyo, Japan) at 25 °C ± 2 °C and at 60% ± 5% residual humidity, until quantifications of atropine were performed. Visual inspection of the sample, atropine sulfate quantification, osmolality and pH measurements, and sterilization tests were carried out immediately after preparation, and at day 14, day 28, and every month until six months for each storage temperature.

Because the prepared eye drops supposed to be stored in the refrigerator and throw it away within one week once eye dropper opened in a clinical setting, the stability of diluted atropine sulfate solution only in unopened multi-dose eyedroppers was examined.

### Atropine sulfate quantification

For each unit, atropine sulfate was quantified using a stability-indicating method adapted previously [5] by liquid chromatography (LC) using the system of LC–MS/MS, which was an UltiMate 3000 HPLC system (Thermo Fisher Scientific K.K., Tokyo, Japan) with an analytical column (Unison UK-C18 column, 50 mm × 3.0 mm, i.d. 3 μm; Imtakt Corporation, Kyoto, Japan). The isocratic mobile phase consisted of a 40:60 (v/v) mixture of 0.1% (v/v) formic acid and acetonitrile. The flow rate of the mobile phase was 0.4 mL/min. The column temperature was set at 25 °C. Mass spectrometric detection was performed on a TSQ Vantage Triple Stage Quadrupole LC/MS Mass Spectrometer (Thermo Fisher Scientific K.K., Tokyo, Japan). The injection volume was 2 μL. The electrospray source was operated in positive mode and mass spectrometer conditions (cone and collision energy) were optimized by direct infusion of the standards. Selected ion monitoring acquisition mode was used for the analysis in order to detect only specific mass ions during the analysis. The MS spectrum of atropine revealed a base peak at m/z 290, corresponding to the pseudo-molecular ions [M + H] ^+^.

To determine the atropine sulfate concentration, 0.1, 1.0, 2.5, and 5.0 mg/mL of atropine sulfate concentration were diluted to 10, 100, 250, and 500 ng/mL by using sterilized water and sterile filtered through 0.2 μm filters (Cosmospin Filter-G; Lot number V8 M3934; Nacalai Tesque, Inc., Kyoto, Japan) to remove any particulate matter.

A calibration curve was prepared using seven concentrations of atropine, i.e., 5, 10, 50, 100, 500, 1000, and 5000 ng/mL, and its linearity was verified three times on other days. If a calibration curve *R*^*2*^ value is greater than 0.999, it is acceptable for determination of atropine using the LC–MS/MS system.

Each day for three days, six solutions of 100 ng/mL were analyzed using a calibration curve prepared the same day. The method’s precision was verified by confirming repeatability that was estimated by calculating the relative standard deviation (RSD) of intra-day analysis. Intermediate precision was assessed using RSD of inter-day analysis. Less than 5% was acceptable for both RSDs. In order to verify the method’s accuracy, recovery of seven defined concentrations to experimental values that were calculated using the mean curve equation was evaluated. With reference to the ICH guidelines, the limit of detection (LOD) and limit of quantification (LOQ) were calculated using equations involving the standard deviation of the response of the curve and the slope of the calibration curve. Because atropine sulfate is chemically stable when stored exposed to light, light susceptibility was not examined [6].

### Visual inspection

Visual inspection of the prepared atropine sulfate solutions was conducted under diffused daylight by transferring the solutions into polycarbonate test tubes. Transparency, color, and presence or absence of visible particles or haziness were verified.

### Osmolality and pH measurements

The pH was measured for each sample using a pH meter (LAQUA D-72 T; Horiba, Kyoto, Japan) equipped with a ToupH® pH electrode which was calibrated at 25 °C in pH 4 and pH 7 buffer solutions (pH standard solution; Horiba, Kyoto, Japan). Osmolality was measured for each solution using a micro-osmometer (Auto 819 Osmomaster®; Biomedical Science Co., Ltd., Tokyo, Japan).

### Sterility assay

The sterility test method was validated using a method adapted from the Japanese Pharmacopoeia sterility assay (4.06), which is harmonized with the European Pharmacopoeia and the U. S. Pharmacopeia [7]. Multi-dose eyedroppers were opened aseptically under a laminar air flow cabinet, and the solutions filtered under vacuum using a Thermo Scientific™ Nalgene® analytical test filter funnel onto a 0.45 μm pore size, 47 mm diameter cellulose nitrate membrane (Whatman®, GE Healthcare, USA). The membranes were rinsed thoroughly using 90 mL of Letheen broth (Difco Laboratories, New Jersey, USA), and rinsed membranes were transferred separately into either a fluid thioglycollate or soy trypticase medium, incubated at 30 °C to 35 °C or 20 °C to 25 °C, respectively, for 14 days, and then examined for the presence of microbial colonies.

### Data analysis—acceptability criteria

The stability of diluted atropine sulfate solutions was verified by evaluating the visual inspections and measuring atropine sulfate concentration, pH, and osmolality.

The study was conducted in accordance with methodological guidelines [8]. Concentration ranged between 90 and 110% of initial concentration (including the limits of a 95% confidence interval of the measures), which was considered as an acceptable level of stability. The observed solutions should be colorless and transparent, with no precipitation. The pH measures and osmolality results were compared with the original preparation (1% atropine sulfate solution).

## Results

### Atropine sulfate quantification

Atropine retention time was 2.6 ± 0.1 min. The calibration curve was found to be linear for concentrations ranging between 5 and 5000 ng/mL and the determined coefficient *R*^2^ was greater than 0.999. The intercept was equal to zero. Recovery of 100 ng/mL was 99.0 ± 0.01, RSD for repeatability was 1.3%, and the intermediate precision RSD was 1.5%. LOD was 0.05 ng/mL and LOQ was 0.5 ng/mL (a signal-to-noise ratio of 19 for an average of six replicates).

### Stability of atropine sulfate in unopened multi-dose eyedroppers

#### Chemical stability

During the experimental period, the mean concentration of six units of atropine sulfate under all conditions was more than 97.8% (Table 1).

**Table 1:** Stability of atropine sulfate concentrations for each storage condition in unopened eyedropper bottles (mean ± 95% confidence interval; *n* = 6)

Set concentration and storage temperature	Initial concentration at Day 0 (mg/mL) (mean ± 95% CI)	Percentage of initial Day 0 measurement (mean ± 95% CI)					
		Day 14	Day 28	Day 60	Day 90	Day 120	Day 180
0.1 mg/mL							
5 °C	0.10 ± 0.32	99.00 ± 1.35	99.65 ± 1.21	98.89 ± 2.14	98.64 ± 1.75	98.45 ± 1.00	98.98 ± 1.19
25 °C		99.98 ± 1.12	98.98 ± 0.41	99.13 ± 0.98	99.31 ± 0.98	98.78 ± 4.00	99.05 ± 3.71
1.0 mg/mL							
5 °C	1.00 ± 0.41	99.01 ± 2.55	99.72 ± 2.15	99.23 ± 0.66	99.18 ± 1.95	98.06 ± 3.45	99.00 ± 1.59
25 °C		100.02 ± 1.38	99.98 ± 0.74	99.65 ± 3.03	99.11 ± 3.09	98.13 ± 4.10	98.55 ± 7.03
2.5 mg/mL							
5 °C	2.50 ± 0.01	99.91 ± 1.01	99.92 ± 1.55	99.84 ± 1.20	100.08 ± 1.88	98.66 ± 6.16	98.99 ± 0.99
25 °C		100.40 ± 2.81	99.94 ± 2.54	99.75 ± 1.33	99.41 ± 4.44	99.15 ± 7.00	98.55 ± 5.33
5.0 mg/mL							
5 °C	5.01 ± 0.02	98.96 ± 6.51	100.10 ± 1.66	103.37 ± 8.90	101.12 ± 3.33	99.85 ± 6.01	97.87 ± 3.68
25 °C		100.05 ± 1.33	99.88 ± 7.00	99.57 ± 0.81	100.02 ± 1.11	98.07 ± 7.06	98.97 ± 0.67

#### Physical stability

Throughout the study, all samples stayed limpid and uncolored, for all examined concentrations and storage conditions, and visible particulate matter or turbidity was not found. Differences in osmolality from the initial value (285 mOsm/kg) were less than 1.40% (4 mOsm/kg) at both storage temperatures (Table 2). The pH did not vary during the experimental period by more than 0.30 and 0.31 pH units from initial pH (5.88) when stored at 5 °C and 25 °C, respectively (Table 3).

**Table 2:** Osmolality change of atropine solutions after storage (mean ± standard deviation; *n* = 6)

Set concentration and storage temperature	Initial osmolality at Day 0 (mOsm/kg) (mean ± SD)	Osmolality values (mean ± SD)					
		Day 14	Day 28	Day 60	Day 90	Day 120	Day 180
0.1 mg/mL							
5 °C	285 ± 0.55	285 ± 3.61	285 ± 2.70	285 ± 1.11	285 ± 1.88	285 ± 0.96	285 ± 1.34
25 °C		285 ± 1.02	285 ± 2.13	285 ± 3.31	285 ± 1.41	285 ± 1.66	285 ± 1.14
1.0 mg/mL							
5 °C	285 ± 0.61	285 ± 1.50	285 ± 3.61	286 ± 3.33	286 ± 2.48	285 ± 0.82	286 ± 2.74
25 °C		286 ± 0.22	285 ± 3.15	286 ± 3.78	285 ± 1.40	285 ± 2.35	286 ± 2.84
2.5 mg/mL							
5 °C	285 ± 0.54	285 ± 0.81	285 ± 2.11	286 ± 3.21	286 ± 2.66	285 ± 0.32	286 ± 3.54
25 °C		284 ± 1.12	286 ± 1.04	286 ± 1.78	286 ± 1.41	286 ± 1.41	286 ± 1.00
5.0 mg/mL							
5 °C	285 ± 0.54	286 ± 1.03	285 ± 2.09	285 ± 0.99	285 ± 0.86	286 ± 1.74	287 ± 0.51
25 °C		286 ± 1.08	283 ± 1.97	286 ± 0.87	286 ± 1.41	287 ± 4.11	286 ± 0.54

**Table 3:** pH change of atropine solutions after storage (mean ± standard deviation; *n* = 6)

Set concentration and storage temperature	Initial pH at Day 0 (mean ± SD)	pH values (mean ± SD)					
		Day 14	Day 28	Day 60	Day 90	Day 120	Day 180
0.1 mg/mL							
5 °C	5.90 ± 0.40	5.91 ± 1.09	5.84 ± 0.15	5.77 ± 1.63	5.69 ± 1.18	5.61 ± 1.16	5.61 ± 1.09
25 °C		5.92 ± 1.06	5.80 ± 1.95	5.70 ± 1.84	5.66 ± 1.27	5.59 ± 1.51	5.61 ± 1.99
1.0 mg/mL							
5 °C	5.88 ± 0.30	5.88 ± 0.99	5.74 ± 0.85	5.74 ± 1.33	5.65 ± 0.14	5.60 ± 0.86	5.58 ± 0.99
25 °C		5.88 ± 1.05	5.70 ± 0.95	5.73 ± 0.74	5.65 ± 0.77	5.60 ± 0.08	5.57 ± 0.70
2.5 mg/mL							
5 °C	5.88 ± 0.31	5.88 ± 0.31	5.74 ± 1.37	5.74 ± 0.79	5.65 ± 0.12	5.60 ± 0.41	5.58 ± 0.39
25 °C		5.88 ± 0.05	5.70 ± 0.61	5.70 ± 0.94	5.65 ± 0.74	5.60 ± 0.12	5.57 ± 0.32
5.0 mg/mL							
5 °C	5.88 ± 0.41	5.88 ± 0.11	5.74 ± 0.39	5.71 ± 0.93	5.70 ± 0.44	5.68 ± 0.15	5.68 ± 0.11
25 °C		5.88 ± 0.47	5.69 ± 0.55	5.68 ± 0.19	5.67 ± 0.32	5.68 ± 0.45	5.68 ± 0.66

### Sterility assay

None of the six analyzed solutions prepared and conserved in unopened bottles at day 0, day 14, day 28, or every month until six months showed any signs of turbidity and hence no evidence of microbial growth when incubated for not less than 14 days at 30–35 °C in case of a fluid thioglycollate medium and at 20–25 °C in the case of a soy trypticase medium.

## Discussion

The diluted atropine sulfate ophthalmic solutions conserved in sterilized polyethylene eyedroppers were physicochemically stable. During six months of experimental observations, mean concentrations of atropine sulfate persisted within a 90 to 110% range of initial concentrations in polyethylene eyedropper bottles that were stored at 5 °C and 25 °C. Additionally, all the solutions that were evaluated in this study decreased no more than 3% by the end of the experiment. Visual aspects and pH did not change at either temperature. Osmolality also was unchanged during the six-month experimental period.

The sterility assay that was conducted following the Japanese Pharmacopoeia revealed no microbial contamination during the experimental period. Antiseptic conditions during medication processing should be maintained for patient safety. Using single-dose eyedroppers is an easy way to achieve microbiological sterility; however, this practice cannot be applied to most hospital compounding departments or community pharmacies in Japan.

According to the package insert, the original 1% atropine sulfate eye drops are described to be stable for at least 36 months before opening and 28 days after opening [9].

Our data have demonstrated that atropine sulfate maintained physicochemical stability at 25 °C for six months without any changes in physical properties when stored in polyethylene eyedropper bottles.

The stability of atropine sulfate has described in the previous studies. Dix J et al. reported that the prepared 0.1% of atropine in 0.9% sodium chloride to treat patients exposed to acetylcholinesterase inhibitors was stable for at least 3 days at ranged 4 °C and 36 °C [10]. Donnelly RF et al. described that 0.1% of atropine in 0.9% sodium chloride were physically compatible and chemically stable when stored for 364 days at 23 °C and exposed to light, or 364 days at 5 °C and protected from light [6]. From these facts, our findings about the chemical stability of diluted atropine solutions are in agreement with the previous studies. Regarding the sterility, the prepared atropine solutions were stored in unopened multi-dose eyedroppers. This storage condition might reduce the risk of microbial contamination. It is necessary that the sterility might not be maintained for a long duration when the product was opened [11].

As with the diluted atropine eye drops, several active pharmaceutical ingredients were prepared as eye drops from the commercially available injections [12–14], eye drops [15] or chemical agents [16]. Most of all cases were antibiotic or antifungal agents, and the stability and sterility were investigated [12–14]. The biological activities, clinical efficacy, safety and pharmacokinetics of these drugs also indicated in some prepared eye drops [15–20]. Despite the increased needs of eye drops in various fields of medicine, many products are still extemporaneously prepared in hospital, and assurance of safety and efficacy of prepared products are needed in each hospital for use. Considering these facts, appropriate medicinal products that answer the needs of clinical settings might be needed.

In this study, atropine sulfate eye drops at 0.1, 1.0, 2.5, and 5.0 mg/mL diluted using 0.9% NaCl that were stored in polyethylene eyedropper bottles were physiologically stable and no obvious physical property change was found during six months of experimental observations.

## Conclusions

This study validated the physical, chemical, and microbiological stability of 0.1, 1.0, 2.5, and 5.0 mg/mL atropine sulfate eye drops conserved inside polyethylene eyedroppers for six months at 25 °C or 5 °C. These information may serve as useful data for preparation of diluted atropine eye drops for pediatric patients.

## Data Availability

None.

## Abbreviations

LC: liquid chromatography
LOD: limit of detection
LOQ: limit of quantification
RSD: relative standard deviation
